# Encouraging Trainees to Write Papers: Is Writing a Case Report the Exclusive Domain of the Attending Physicians?

**DOI:** 10.31662/jmaj.2023-0029

**Published:** 2023-05-12

**Authors:** Shigeki Matsubara, Alan Kawarai Lefor

**Affiliations:** 1 Department of Obstetrics and Gynecology, Jichi Medical University, Tochigi, Japan; 2 Department of Obstetrics and Gynecology, Koga Red Cross Hospital, Koga, Japan; 3 Department of Surgery, Jichi Medical University, Tochigi, Japan

**Keywords:** attending doctor, publication, responsibility, trainee, writing papers

## Dear Editors:

As per Saeki’s view ^(1)^, publications from Japan are declining in number and encouraging trainees to write may increase productivity. With decades-long experience writing papers and as department chair, we have proposals, which have not been discussed previously. Changing the tradition regarding “who writes a paper” and strengthening the senior staff’s responsibility may increase productivity.

A case report is often written by trainees of patient’s own attending (referred to as “attending” (tanto-i) hereafter). Senior staff can encourage them to write and offer help; however, if attending trainees lack personal motivation to write “now”, forcing them to write is difficult. In such cases, the senior staff must decide who in the department will write, under the condition that the physician understands a patient’s course and significance, regardless of whether he/she is a trainee or an “attending”. Depending entirely on the attending may lead to valuable cases not being reported, which would thus be lost forever. Reporting a unique patient is doctor’s/department’s responsibility ^(2)^ beyond who writes it.

The senior staff should be willing and prepared to write. However, one may be concerned that physicians other than the attending writing papers may lead to a bad departmental atmosphere. In such situations, the senior staff should write the paper as first author. As per the Japanese tradition, professors do not write first-authored case reports, which is peculiar. A case report is no less important than an original article, since the significance matters more than the type of the article. If a speedy publication is required and for some reason the trainee is unable to start, immediate writing should become the responsibility of the senior staff.

Productivity discussions should be divided, future versus now. Educating a trainee is the senior staff’s responsibility, which can be seen as a future investment. Junior attendings are the most suitable individuals for writing. However, making it is essential to create an atmosphere where anybody can write, regardless if they are the patient’s own attending or not. This may help increase productivity. Taking time to educate or to write immediately, senior staff must decide which strategy is the best on a case-by-case manner. Experience is not the exclusive possession of attendings but a departmental or medical society resource.

## Article Information

### Conflicts of Interest

None

### Author Contributions

S. Matsubara: Identification of the significance and manuscript writing. AKL: Manuscript editing. The authors are responsible for the conceptualization and the writing of the manuscript.

### Approval by Institutional Review Board (IRB)

Not applicable

### Patient Anonymity

Not applicable

### Informed Consent

Not applicable
